# Multilevel analysis of factors for intimate partner violence during pregnancy in Gammo Goffa Zone, South Ethiopia: A community based study

**DOI:** 10.3389/fpubh.2023.1122041

**Published:** 2023-03-14

**Authors:** Mesfin Mamo Utaile, Ahmed Ali Ahmed, Alemayehu Worku Yalew

**Affiliations:** ^1^Department of Public Health, College of Medicine and Health Sciences, Arba Minch University, Arba Minch, Ethiopia; ^2^Department of Preventive Medicine, School of Public Health, College of Health Sciences, Addis Ababa University, Addis Ababa, Ethiopia

**Keywords:** intimate partner, violence, pregnancy, mixed-effect, Ethiopia

## Abstract

**Background:**

Intimate partner violence during pregnancy is a public health problem that can affect both maternal and fetal life. However, its prevalence and associated factors have not been well studied and understood in Ethiopia. Hence, this study was conducted to assess the individual and community-level factors associated with intimate partner violence during pregnancy in Gammo Goffa Zone, South Ethiopia.

**Methods:**

A community-based cross-sectional study was conducted among 1,535 randomly selected pregnant women from July to October 2020. Data were collected using an interviewer-administered, standardized WHO multi-country study questionnaire and analyzed using STATA 14. A two level mixed-effects logistic regression model was used to identify factors associated with intimate partner violence during pregnancy.

**Results:**

The prevalence of intimate partner violence during pregnancy was found to be 48% (95% CI: 45–50%). Factors affecting violence during pregnancy were identified at the community and individual levels. Access to health facilities (AOR = 0.61; 95% CI: 0.43, 0.85), women feeling isolated from the community (AOR= 1.96; 95% CI: 1.04, 3.69), and strict gender role differences (AOR= 1.45; 95% CI: 1.03, 2.04) were among higher-level factors found to be significantly associated with intimate partner violence during pregnancy. Low decision-making power was found to increase the odds of experiencing IPV during pregnancy (AOR= 2.51; 95% CI: 1.28, 4.92). Similarly, maternal education, maternal occupation, living with the partner's family, current pregnancy intended by the partner, dowry payment, and presence of marital conflict were among the individual- level factors found to increase the odds of experiencing intimate partner violence during pregnancy.

**Conclusions:**

The prevalence of intimate partner violence during pregnancy was high in the study area. Both individual and community-level factors had significant implications on maternal health programs related to violence against women. Socio-demographic and socio-ecological characteristics were identified as associated factors. Since it is a multifaceted problem, special emphasis has to be given to multi-sectoral approaches involving all responsible bodies to mitigate the situation.

## Introduction

Violence against women (VAW) is one of the most pervasive and least addressed human rights violations, derived from unequal power relationships between men and women (1). VAW is defined based on the type of violent act or the relationship between the victim and perpetrator (2, 3). The World Health Organization (WHO) defines VAW as “any act of gender-based violence that results in or is likely to result in, physical, sexual, or psychological harm or suffering to women, including threats of such acts, coercion, or arbitrary deprivation of liberty, whether occurring in public or private life” (4). The most common form of VAW is intimate partner violence (IPV). IPV can occur at any stage, even during pregnancy (4). IPV during pregnancy is a worldwide problem that affects the health of women and their fetuses, with a high burden in developing countries (5).

Globally, IPV during pregnancy is considered an important public health problem because of its connection with adverse maternal and fetal outcomes (6). It might occur both in conflict-or crisis-affected and in more stable ones (7). Addressing IPV during pregnancy is crucial to the achievement of international and national goals related to maternal health (8).

Whether a woman is at increased risk for IPV during pregnancy is a controversial issue among researchers. However, it is a significant public health problem, with rates varying considerably by country and maternal risk factors globally (5, 9). The overall global estimates of IPV against pregnant women vary between 3 and 30% (10), with higher prevalence reported in resource-poor countries (3, 11, 12). Africa is the region with the highest prevalence of IPV during pregnancy. In Africa, the magnitude of IPV against pregnant women is between 2 and 57%, with meta-analysis yielding a pooled estimate of 15.23% (13). Prior studies in Ethiopia indicated various rates on the prevalence of pregnancy-related IPV ranging from 12 to 36% (5, 14–17).

IPV during pregnancy is associated with multiple factors. It is a problem not caused by any single factor. Rather, it is caused by a combination of several factors that may increase the likelihood of a man perpetrating violence and the risk of a woman becoming a victim (18). The majority of previous studies focused on individual-level factors associated with IPV during pregnancy, such as childhood inter-parental exposure, early marriage, dowry payment, residence, alcohol use, and/or education (5, 16, 17). Therefore, it is critical to consider violence as a multifaceted event involving interactions both at the community and individual levels (5). It is also suggested by WHO that gender inequality and norms on the acceptability of violence against women are the root causes of violence against women (12).

IPV has a significant negative impact on women's health and quality of life (3, 12, 19). Those adverse consequences are amplified in pregnancy, with an increased risk of pregnancy outcomes, such as preterm birth, low birth weight, and small for gestational age (7, 20). It can also serve as a predisposing factor for maternal morbidity and mortality, resulting mainly from its physical, mental, and reproductive health consequences (10, 21).

Studies in Ethiopia have serious limitations in evaluating the determinants of IPV during pregnancy. Most of the studies generated evidence from facility-based surveys (5, 15–17, 22, 23), except one community-based cross-sectional study conducted on determinants of IPV during pregnancy among married women in Abay Chomen District, Western Ethiopia (24). Addressing the problem at the facility level is likely to present an inadequate image of IPV during pregnancy. Furthermore, the nesting effects of different levels were disregarded in the previous studies (5, 15–17, 22–24). Therefore, a multilevel epidemiological study is required for a more comprehensive understanding of the problem. Consequently, all potential determinants of IPV during pregnancy can be assessed to propose maternal service strategies related to IPV during pregnancy at different levels. Thus, this community-based study was conducted to assess the effect of individual and community-level factors on the occurrence of IPV during pregnancy. Moreover, it is the baseline assessment of an ongoing cohort to identify the effect of IPV on maternal and neonatal outcomes.

## Materials and methods

### Study design and setting

A community-based cross-sectional study was conducted in the Gammo Goffa Zone between July and October 2020. Gammo Goffa Zone is one of the 14 zones of the Southern Nations, Nationalities, and Peoples Regional (SNNPR) State of Ethiopia. Its capital, Arba Minch, is located 505 km south of Addis Ababa, and 275 km southwest of Hawassa, the capital city of the region. Administratively, the zone is subdivided into 15 rural districts designated as ‘Woredas' and two town administrations. According to the population projection of Ethiopia for all regions at the woreda level from 2014–2017, the zone had a total population of 2,043,668 (25).

### Population, sample size, and sampling procedure

In this cross-sectional study, pregnant women were the study population. The required sample size was determined using a single population proportion formula based on the following assumptions: a 35.6% prevalence of IPV during pregnancy in Ethiopia (5), a 95% level of the confidence interval, and a 4% degree of precision. Due to the multistage cluster sampling method used, a design effect of 2 was considered. Finally, 10% was added to the non-response rate. Accordingly, the final sample size was calculated to be 1,210. However, this study was a baseline survey of a cohort study to determine the effect of IPV during pregnancy on maternal and neonatal health outcomes, in which 1,535 pregnant women were followed up. Thus, to increase the precision of the estimates and the power of the study, the sample size was increased to 1,535. The sample size is adequate to identify factors associated with IPV during pregnancy.

**Figure 1:**
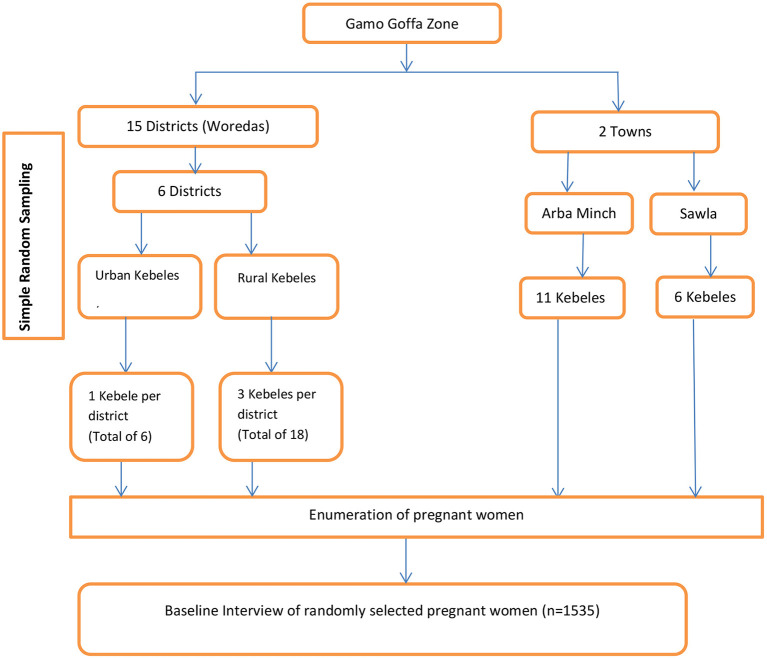
Schematic presentation of sampling procedure for the cross sectional study on IPV during pregnancy.

A multi-stage cluster sampling technique was employed to identify the study participants. Initially, the zone was stratified in to town administrations and rural districts. Then, in the first stage, by considering time and logistics, six districts were selected randomly. In the second stage, all the selected districts were stratified into urban and rural *kebeles*. A kebele is the smallest administrative unit (in the government structure) that is considered a cluster in this study. Then, 3 rural *kebeles* and 1 urban *kebele* were randomly selected from each selected district. In this zone, there were two town administrations (Arba Minch and Sawla) with 11 and 6 *kebeles* respectively, and all were purposefully included. A total of 41 clusters were selected randomly. Then, at the household level, an enumeration of pregnant women was conducted in the selected *kebeles* to fix a sampling frame. After identifying households with pregnant women, proportional to sample size allocations were employed. Finally, a simple random sampling was carried out to identify respondents from the selected households as a study unit (Figure 1).

### Study variables and measurements

The dependent variable for this study was IPV during the current pregnancy. IPV during the current pregnancy was defined as the experience of at least one act of any form of violence (psychological, physical, or sexual violence) by women perpetrated by their current or most recent partners, during the current pregnancy period.

Psychological violence was measured as the experience of one or more acts or threats of acts, such as (a) being insulted, (b) being humiliated, (c) being intimidated, or d) threatening to hurt the study participant or someone the study candidate cares about (3).

Physical violence was defined as the experience of one or more acts of physical aggression, such as (a) being slapped or having something thrown at her that could hurt her, (b) being pushed or shoved, (c) being hit with a fist or something else that could hurt; (d) being kicked, dragged, or beaten up; (e) being choked or burned on purpose, and/or (f) being threatened with, or actually having, a gun, a knife, or another weapon used on her by an intimate partner (3).

Sexual violence was measured as the experience of one or more acts, such as a) being physically forced to have sexual intercourse, when she did not want to, b) having sexual intercourse because she was afraid of what her partner might do, and/or c) being forced to do something sexual that she found humiliating or degrading to her by an intimate partner (3).

The independent variables were divided into two levels. Level-1 (lower-level variables), included individual and household characteristics, such as socio-demography, wealth index, reproductive and obstetric characteristics, women's autonomy, and partnership-related variables. The wealth index was computed using principal component analysis (PCA). Level 2 (higher-level) variables included community and societal characteristics, such as place of residence, access to health facilities, and socio-ecological factors. The independent variables were selected based on their relationship with the dependent variable identified through reviewing existing literature (3, 15–17). In the previous studies, they were described as existing at one level; in this study, they are identified as variables operating at different levels.

### Data collection

A pre-tested interviewer-administered structured questionnaire was adapted from the WHO multi-country study of the VAW questionnaire (3). The indicators for the wealth index were adapted from EDHS (26). The questionnaire was prepared in English and translated to the local language (Amharic), and back-translated to English by another person to ensure its consistency and accuracy. Health extension workers were recruited, trained and deployed for data collection. The data collection process was supervised by trained supervisors and principal investigators. The data collectors and supervisors were recruited based on their eloquence in local languages, qualifications, and experience in data collection. The WHO's practical guide for researching VAW was adopted and used by the research team (27). Furthermore, we didn't encounter any disruption during the study period due to COVID-19 because there was no strict lockdown/shutdown in Ethiopia and the disease incidence was very slow.

### Data analysis

After the data were coded and entered into EpiData v 3.1, were exported to STATA 14 for cleaning, editing, and analysis. Descriptive statistics were computed and presented. Socioeconomic quintiles were determined using principal component analysis (PCA).

Since the occurrence of IPV during pregnancy is affected at different levels, a mixed-effects multilevel logistic regression model was employed. A bivariate analysis was done using cross-tabulation to test the association between IPV during pregnancy and independent variables. All variables having *P* < 0.25 were considered candidates for the final model. In this analysis, a two-level binary logistic regression model was used. The individual and family-level characteristics were considered as lower-level variables, and the community and societal characteristics were treated as higher-level variables. Generally, two models were estimated. These were the intercept-only model; an empty model, that contained no covariates, and a full model that included lower-level (level-1) and higher-level (level-2) variables. The goodness of fit of the multilevel model was tested by the log-likelihood ratio (LR) test. Multicollinearity between independent variables was assessed, using the variance inflation factor (VIF).

### Ethical considerations

The study was approved for scientific and ethical integrity by the Research and Ethical Review Committee (RER) of the School of Public Health, the Institutional Review Board (IRB) of the College of Health Sciences, Addis Ababa University (Protocol number: 106/19/SPH). Written informed consent was sought from every study participant. For women under the age of 18, consent was obtained from their parents. The study strictly followed the WHO guidelines on ethical issues related to violence research (28).

All interviews were conducted in complete privacy. Data collectors were instructed to refer women with serious psychological distress to health facilities and act accordingly. After the completion of interviews, data collectors were observed for 14 days. The data collectors wore protective face masks. A reasonable physical distance was kept between interviewers and interviewees during data collection.

## Results

### Socio-demographic characteristics

A total of 1,535 pregnant women were included in the study. About three-fifths (58.0%) were urban residents. The majority, 1,332 (86.8%), were in the age group of 20- 34, with a mean (±SD) age of 26.3 ± 4.7 years. Most of the respondents (95.4%) were married at the time of the interview. In terms of ethnicity and religion, study participants were predominantly Gammo (57.7%) and protestant Christians (67.6%). Nearly half of the respondents (45.6%) attended primary school. The majority of them, 1035 (67.4%), were housewives. Nearly half of the partners/husbands, 748 (48.7%), were in the age group of 25–34, with a mean (±SD) age of 33.8 ± 6.7 years, and ranging from 18 to 56 years. More than one-third (33.2%) of partners attended grades 9–12, and farming was the leading occupation of their partners, with 674 (43.9%) (Table 1).

**Table 1:** Socio-demographic characteristics of pregnant women and their partners in Gammo Goffa Zone, South Ethiopia, July to October, 2020, (*n* = 1,535).

**Variables**	**Number**	**Percent**
**Residence**		
Urban	891	58.0
Rural	644	42.0
**Age of respondent (in years)**		
15–19	104	6.8
20–34	1,332	86.8
35–49	99	6.4
**Ethnicity**		
Gammo	886	57.7
Goffa	400	26.1
Others	249	16.2
**Religion**		
Protestant Christian	1,037	67.6
Orthodox Christian	437	28.5
Others	61	4.0
**Marital status**		
Married	1,464	95.4
Cohabited	46	3.0
Widowed/separate/divorced	25	1.6
**Educational status**		
No formal education	331	21.6
Primary (1–8)	700	45.6
Secondary (9–12)	338	22.0
Tertiary (12^+^)	166	10.8
**Occupational status**		
Housewife	1,035	67.4
Employed (GO/NGO/Private)	252	16.4
Others	248	16.2
**Wealth quintile**		
Poorest	308	20.1
Poor	306	19.9
Middle	243	15.8
Rich	383	25.0
Richest	295	19.2
**Partners' age**		
18–24	91	5.9
25–34	748	48.7
35–49	629	41.0
>50	67	4.4
**Partners' educational status**		
No formal education	193	12.6
1–8	345	22.5
9–12	510	33.2
>12	487	31.7
**Partners' occupation**		
Employee	416	27.1
Farmer	674	43.9
Merchant	238	15.5
Other	207	13.5

### Reproductive and behavioral characteristics

The majority, 1137 (74.1 %), of respondents were married at the age of 18 or above, with the mean age of their first marriage being 20.72 years (±3.1 SD). Two hundred seventeen (14.1%) couples began living together without having a marriage ceremony. Only 136 (8.9%) of respondents did not volunteer to marry their current partner. Likewise, 339 (22.1%) of them had received dowry/bride price payments. Most (89.3%) of the study participants reported that the current pregnancy was intended by their partners. In this study, more than 90% of the respondents never used khat or alcohol. The majority (90.2%) of respondents had partners who never chewed khat. More than one-third of pregnant women, 516 (33.6%), reported that their intimate partners used alcoholic drinks (Table 2).

**Table 2:** Reproductive and behavioral characteristics of pregnant women in Gammo Goffa Zone, South Ethiopia, July to October, 2020, (*n* = 1,535).

**Variables**	**Number**	**Percent**
**Age at first marriage (in years)**		
< 18	398	25.9
>18	1,137	74.1
**Level of marriage**		
First	1,432	93.3
Second	90	5.9
Third and above	13	0.8
**Marriage ceremony**		
Religious	616	40.1
Customary	568	37.0
Civil	134	8.7
No ceremony	217	14.1
**Dowry/bride price payment**		
No	1,196	77.9
Yes	339	22.1
**Volunteer to marry**		
Yes	1,399	91.1
No	136	8.9
**Pregnancy intended by partner's**		
Yes	1,370	89.3
No	165	10.7
**Respondent chew khat**		
No	1,464	95.4
Yes	71	4.6
**Respondent drink alcohol**		
No	1,403	91.4
Yes	132	8.6
**Partner chew khat**		
No	1,384	90.2
Yes	151	9.8
**Partner drink alcohol**		
No	1,019	66.4
Yes	516	33.6

### Prevalence and forms of IPV during pregnancy

The occurrences and forms (psychological, physical, and sexual) of IPV during the current pregnancy were assessed. Accordingly, the overall prevalence of IPV during the current pregnancy was found to be 48%, (95% CI: 45–50%). The joint occurrences of different forms of IPV were also evaluated. Therefore, 26.3, 24.6, and 1.4% of women experienced psychological, physical, and sexual violence in isolated form, respectively. The overlapping occurrences of psychological and physical violence account for 11.8%. The other two forms, such as psychological plus sexual violence and physical plus sexual violence account for 6.1 and 1.3%, respectively. One in every three (31.7%) women experienced multiple forms (psychological + physical + sexual) of IPV during their current pregnancy (Table 3).

**Table 3:** Prevalence of IPV among pregnant women in Gammo Goffa Zone, South Ethiopia, July to October, 2020, (*n* = 1,535).

**Forms of IPV**	**Number**	**Percent**
**Psychological violence**		
Insulted/made to feel bad	409	26.6
Humiliated/belittled	109	12.4
Intimidated/scared on purpose	111	7.2
Threatened/hurt	41	2.7
**Any act of psychological v**	**531**	**34.6**
**Physical violence**		
**Moderate physical violence**	**461**	**30.0**
Slapped /thrown some thing	344	22.4
Pushed/shoved	184	12.0
**Severe physical violence**	**170**	**11.1**
Hit with fist or something	96	6.3
Kicked/dragged/beaten	54	3.5
Choked/burnt	33	2.1
Threatened/used weapon	22	1.4
**Any act of physical violence**	**524**	**34.1**
**Sexual violence**		
Physically forced to have sex	234	15.2
Having sex due to fear	134	8.7
Forced to do humiliating sex	36	2.3
**Any act of sexual violence**	**297**	**19.3**
**Any form of IPV**	**735**	**48.0**

Bold values indicate the prevalence of each form of violence.

### Psychological violence during pregnancy

During the current pregnancy, 26.6% of women were verbally insulted by their intimate partners. Among the study participants, 12.4% of pregnant women were belittled in front of others during their recent pregnancy. Likewise, in their pregnancy, 7.2% were intimidated and 2.7% were threatened at least once by their intimate partner. Generally, the prevalence of intimate partner psychological violence during the current pregnancy was 34.6%, with a 95% CI of 32.0–37.0% (Table 3).

### Physical violence during pregnancy

In this study, the types of physical acts that abused women experienced during their current pregnancy were assessed. Among those who experienced physical violence, 334 (22.4%) were slapped and 184 (12.0%) were pushed or shoved by their intimate partners. The percentage of women who were hit with a fist by a partner was found to be 6.3%. Though classifying acts of physical violence by severity is debatable (3), 461 (30.0%) of pregnant women experienced acts of severe physical violence (hit with a fist, kicked, dragged, or threatened with a weapon), whereas 170 (11.1%) of them reported acts of moderate physical violence (slapped, pushed, or shoved). Generally, the prevalence of intimate partner physical violence during the current pregnancy was 34.0%, with a 95% CI of 31.8–36.5% (Table 3).

### Sexual violence during pregnancy

The proportion of women physically forced to have sexual intercourse against their will was 15.2%. About 9% of pregnant women reported that they had sexual intercourse due to fear of their partners. Moreover, 36 (2.3%) of respondents were coerced by their partners into sexual practices that they found degrading or humiliating. In general, 297 (19.3%, 95% CI of 17.4–21.4%) women have reported at least one act of sexual violence during their current pregnancy (Table 3).

### Factors associated with IPV during pregnancy

In this study, we used multilevel models to investigate the cluster variance of IPV exposure during pregnancy. To determine whether there was any variation in experiencing IPV during pregnancy between clusters (kebeles) and to decide on the evaluation of the random effects at the cluster level, the intra-class correlation coefficient (ICC) was calculated in the null model and it was found to be 0.229, indicating that 22.9% of the total variance was contributed by between cluster variations. Finally, after ensuring that IPV during pregnancy was clustered significantly by kebele (*p* < 0.0001), the full model was run by including higher-level and lower-level variables. The ICC then decreased to 0.207, indicating that cluster-level variables accounted for 20.7% of the total variance, signifying a preference for multilevel analysis. The test of the preference of log-likelihood vs. logistic regression was still strongly significant (*P* < 0.0001) (Table 4).

**Table 4:** Parameter coefficients and test of goodness-of-fit of the multilevel model, in Gammo Goffa Zone, South Ethiopia, July to October, 2020, (*n* = 1,535).

**Models**	**Fixed intercept-cons (95 % CI)**	**Random effect as level-2 variance**	**Intra-class correlation coefficient: ICC (ρ)**	**Log likelihood (LR) (deviance)**	**Significance of LR test vs. logistic regression (*P*-value)**
		**var [-cons (95 % CI)]**			
Null model	−0.01 (−0.33, 0.31)	0.98 (0.57, 1.68)	0.229 = 22.9%	−970.60	< 0.0001
Full model	1.31 (0.69, 2.47)	0.86 (0.49, 1.49)	0.207 = 20.7%	−934.13	< 0.0001

In the final two-level binary logistic regression model, both cluster-level variables and individual-level variables were found to be important factors associated with IPV during pregnancy. Among the cluster-level variables, access to health facilities, socio-ecological factors such as women feeling isolated from the community, and strict gender role differences between men and women in society were found to have a statistically significant association with IPV during pregnancy. Pregnant women who were from clusters (kebeles) found within 2 h of travel on foot from health facilities (hospital/health center) (AOR = 0.61; 95% CI: 0.43, 0.85) had a significant association with the experience of IPV during pregnancy. The odds of experiencing IPV during pregnancy among women who were feeling isolated from the community were about two times higher (AOR= 1.96; 95%: 1.04, 3.69) than among women who were not feeling isolated from the community. Similarly, the presence of strict gender role differences between men and women in society (AOR= 1.45; 95% CI: 1.03, 2.04) was found to be significantly associated with IPV during pregnancy.

Among the socio-demographic and economic characteristics considered at level-1, educational status, occupational, living with a partner's family and women's decision-making power at home were found to have a statistically significant association with the occurrence of IPV during pregnancy. Women who attended primary (AOR = 0.69; 95%CI: 0.50, 0.97), secondary (AOR = 0.66; 95%CI: 0.45, 0.97), or tertiary (AOR = 0.01; 95%CI: 0.00, 0.03) had lower odds of experiencing IPV during pregnancy as compared to women who didn't attend any formal education. Employed women (AOR = 2.68; 95%CI: 1.68, 4.25), and those women who lived with their husband's families (AOR = 1.57; 95%CI: 1.10, 2.22) had a higher risk of IPV during pregnancy than their counterparts. Similarly, the odds of experiencing IPV during pregnancy among women with low decision-making power at home were 2.5 times higher than those women with high decision-making power (AOR = 2.51; 95%CI: 1.28, 4.92).

Partner-intended pregnancy and dowry payment, or “bride price,” were among the individual-level reproductive factors found to have a significant association with the occurrence of IPV during pregnancy. Women with a current pregnancy intended by their partners (AOR = 0.62; 95%CI: 0.41, 0.93) were less likely to experience IPV during pregnancy when compared with women with a current pregnancy not intended by their partners. Compared to women who had no dowry payments, women who had dowry payments (AOR = 1.42; 95% CI: 1.03, 1.96) appeared to experience significantly higher levels of IPV during pregnancy.

Among the socio-ecological factors considered at the individual-level, having rejecting father during childhood, a partner's experience of violence as a child, a partner having rejecting father during childhood, and having difficult communication with a partner had a non-significant association with IPV during pregnancy. However, the presence of marital conflict had a significant association. Women with a marital conflict (AOR = 1.56; 95%CI: 1.13, 2.16) had higher odds of experiencing IPV during pregnancy when compared to those without a marital conflict (Table 5).

**Table 5:** Multilevel analysis of factors affecting IPV during pregnancy, Gammo Goffa Zone, South Ethiopia, July to October, 2020, (*n* = 1,535).

**Variables**	**IPV during current pregnancy**		
	**N (%)**	**COR (95% CI)**	**AOR (95% CI)**
**Level-1 variables**			
*Individual and household characteristics*			
**Wealth quintiles**			
First quintile	171 (55.5%)	1.00	1.00
Second quintile	165 (53.9%)	0.94 (0.68, 1.29)	1.01 (0.64, 1.61)
Third quintile	114 (46.9%)	**0.71 (0.51, 0.99)**	1.14 (0.67.1.91)
Fourth quintile	158 (41.3%)	**0.56 (0.42, 0.76)**	0.91 (0.55, 1.50)
Fifth quintile	127 (43.1%)	**0.61 (0.44, 0.84)**	0.67 (0.40, 1.12)
**Educational status**			
No formal education	203 (61.3%)	1.00	1.00
1–8	363 (51.9%)	**0.68 (0.52, 0.88)**	0.69 (0.50, 0.97)^*^
9–12	164 (48.5%)	**0.59 (0.41, 0.81)**	0.66 (0.45, 0.97)^*^
>12	5 (3.0%)	**0.02 (0.01, 0.05)**	0.01 (0.00, 0.03)^*^
**Occupational status**			
House wife	474 (45.8%)	1.00	1.00
Employee (GO, NGO, and Private)	126 (50.0%)	1.18 (0.89, 1.56)	2.68 (1.68, 4.25)^*^
Others (daily laborers, housemaid)	135 (54.4%)	**1.41 (1.07, 1.87)**	1.12 (0.77, 1.62)
**Living with husband's family**			
No	580 (45.9%)	1.00	1.00
Yes	155 (57.0%)	**1.56 (1.20, 2.03)**	1.57 (1.10, 2.22)^*^
**Substance use (Khat/alcohol)**			
No	692 (47.3%)	1.00	1.00
Yes	43 (60.6%)	**1.71 (1.05, 2.79)**	1.37 (0.69, 2.74)
**Partner occupation**			
Employee	210 (50.5%)	1.00	1.00
Farmer	299 (44.4%)	**0.78 (0.61, 0.99)**	1.05 (0.72, 1.52)
Merchant	132 (55.5%)	1.22 (0.88, 1.68)	1.38 (0.86, 2.21)
Other	94 (45.4%)	0.82 (0.58, 1.14)	1.12 (0.67, 1.86)
**Women's decision making power at home**			
High	689 (46.7%)	1.00	1.00
Low	46 (75.4%)	**3.94 (1.93, 6.31)**	2.51 (1.28, 4.92)^*^
***Reproductive characteristics***			
**Pregnancy intended by partner's**			
No	102 (61.8%)	1.00	
Yes	633 (46.2%)	**0.53 (0.38, 0.74)**	0.62 (0.41, 0.93)^*^
**Dowry/bride price**			
No	538 (45.0%)	1.00	1.00
Yes	197 (58.1%)	**1.70 (1.33, 2.17)**	1.42 (1.03, 1.96)^*^
***Socio-ecological characteristics***			
**Women had rejecting father during childhood**			
No	662 (46.5%)	1.00	1.00
Yes	73 (65.8%)	**2.21 (1.47, 3.32)**	1.57 (0.94, 2.59)
**Partner experienced violence as a child**			
No	661 (46.9%)	1.00	1.00
Yes	74 (58.7%)	**1.61 (1.11, 2.33)**	1.11 (0.67,1.85)
**Partner had rejecting father during childhood**			
No	640 (46.5%)	1.00	1.00
Yes	95 (59.7%)	**1.71 (1.22, 2.38)**	1.27 (0.79,2.04)
**Marital conflict**			
No	453 (44.2%)	1.00	1.00
Yes	282 (55.2%)	**1.55 (1.25, 1.92)**	1.56 (1.13, 2.16)^*^
**Difficult communication with partner**			
No	593 (46.4%)	1.00	
Yes	142 (55.0%)	**1.41 (1.08, 1.85)**	0.85 (0.58, 1.23)
**Level-2 variables**			
*Communal (kebele) characteristics*			
**Average distance from health facilities (Hospital/H center)**			
< 2 h	440 (50.6%)	1.00	1.00
>2 h	295 (44.3%)	**0.78 (0.63, 0.95)**	0.61 (0.43, 0.85)^*^
*Socio-ecological characteristics*			
**Isolated from the community**			
No	679 (46.5%)	1.00	1.00
Yes	56 (73.7%)	**3.22 (1.91, 5.42)**	1.96 (1.04, 3.69)^*^
**Violence as a means to settle conflict**			
No	587 (46.1%)	1.00	1.00
Yes	148 (56.7%)	**1.53 (1.17, 2.01)**	1.29 (0.87, 1.92)
**Strict gender role differences**			
No	538 (44.9%)	1.00	1.00
Yes	197 (58.5%)	**1.73 (1.35, 2.21)**	1.45 (1.03, 2.04)^*^

* Significant at a *p*-value of < 0.05, COR, Crude odds ratio; AOR, Adjusted odds ratio; CI, confidence interval.

Bold values indicate the significance of associations.

## Discussion

In the study, the overall prevalence of IPV during pregnancy was found to be 48%, which is relatively consistent with a previous study conducted in Abay Chome, West Ethiopia (24). Nevertheless, the current finding is lower than the findings of prior studies in Gonder and Addis Ababa, Ethiopia (22, 29). However, it was higher than previous findings from other parts of the country (5, 15, 16, 30) and other African countries, where IPV during pregnancy ranged from 17 to 31% (31–35). Those variations might be due to differences in study areas, study designs, and socio-cultural differences among study settings, including community norms, beliefs, customs, and traditions related to gender issues. Furthermore, this finding can indicate that the level of IPV during pregnancy is still unacceptably high in an African setting.

Unlike other studies conducted in Ethiopia (22, 24), the most common form of IPV reported in this study was psychological violence (34.6%), followed by physical violence (34.0%) and sexual violence (19.3%). This is almost comparable with findings reported elsewhere (32, 33). However, the prevalence of psychological IPV reported in this study was lower than findings in countries like Nepal and Kenya (32, 36), where their prevalence was found to be 53.8 and 55.8%, respectively. This variation might be due to traditional gender norms that support violence in the respective communities (5).

Moreover, the joint occurrence of psychological and physical violence was the most common compared to the other two occurrences (psychological plus sexual and physical plus sexual). This is similar to a finding from West Wollega, Western Ethiopia (37). This might explain why physical violence is often complemented by psychological abuse (3). The overlap of physical and sexual violence in our study was lower than in the study in Abay Chome, West Ethiopia (24). That might be due to differences in perception of violence and the study population in the respective settings.

In this multilevel epidemiological study, both the higher-level and lower-level variables were found to be factors associated with IPV during pregnancy, suggesting the need for interventions both at the community and individual levels. Among the higher (cluster) level variables, access to health facilities, socio-ecological factors, such as women feeling isolated from the community, and strict gender role differences between men and women in society were the important determinants of IPV during pregnancy. This is in agreement with the finding of the study conducted in Jimma, Ethiopia (5). This may be explained by the fact that women living in communities where victims of IPV are isolated may have an increased risk of developing IPV, due to the fact that the communities may share similar attitudes and perceptions and more readily accept IPV. Additionally, the presence of strict gender role differences between men and women may support marked inequalities between men and women and can lead to tolerance and acceptance of IPV during pregnancy (38).

Among the socio-demographic and economic characteristics considered as level-1 variables, educational status, occupation, living with a partner's family, and women's decision-making power at home were identified as determinants of IPV during pregnancy. Women with higher education levels were less likely to experience IPV during pregnancy. This finding is consistent with other prior studies (37, 39, 40). It is also supported by a meta-analysis conducted among pregnant women in Ethiopia (30). Therefore, it may be explained by the fact that improving literacy may help to minimize the occurrence of IPV in pregnancy. It may also be explained that educated women can negotiate for greater autonomy and control of resources, which may reduce their risk of experiencing violence (3).

Contrary to other studies (23, 37, 41), employed women were more likely to experience IPV than unemployed (housewife) women in this study. This might be explained by the fact that employed women might have more exposure to see how people are reacting against human rights violations in workplaces. That may enable them to report any violation of rights, including IPV during pregnancy. This can also be explained by the fact that men in a conservative society might be violent toward employed women as a means of controlling them. Moreover, women's economic empowerment may serve as an obstacle to power-sharing with men, which may lead to the perpetration of violence against women (42).

In this study, the likelihood of experiencing IPV during pregnancy was higher among women with low autonomy. This finding is supported by evidence from other studies (15, 37, 43). However, this finding is in contrast to the findings of other studies conducted in African countries, where empowered women were more likely to experience IPV compared to their counterparts (44, 45). A possible explanation for the discrepancies may be differences in the socio-cultural context. Moreover, making women autonomous is one of the preventive factors against IPV. Women with high autonomy (empowered women) can negotiate for their rights and do not accept men's dominance, which could possibly serve as a means to prevent the occurrence of IPV during pregnancy (43). The current finding further suggests advocating for the prevention of IPV against women that may occur due to low autonomy.

In contrast to a prior study conducted in the country, women not living with their partner's family appear to experience significantly lower levels of IPV during pregnancy compared to women living with their partner's family (24). This can be explained by the fact that women living with their partner's family in the Ethiopian context are expected to serve and respect the parents of their husbands. This in turn gives a favorable environment to those parents to escalate social norms and notions of masculinity associated with power and dominance that may lead to men perpetrating violence against women.

Among reproductive characteristics considered level-1 variables, the partner's intention of current pregnancy and dowry payment were found to be important determinants of IPV during pregnancy. In this study, intended pregnancy had a significant association with IPV during pregnancy. This is consistent with other previous studies (17, 46). This may be due to the fact that an intended pregnancy may reduce conflict among couples, reducing violence. The current finding may also suggest the importance of working on male involvement in planning successive pregnancies. This may ultimately reduce the occurrence of IPV during pregnancy. Dowry payment was found to increase the risk of IPV during pregnancy. This is comparable with another prior study conducted in the country (24). That could be attributed to the fact that the bride price is a payment made to the bride's family, which may lead to disagreements among couples and result in violence against women. In developing countries like Ethiopia, where most people reside in rural settings, dowry payment is the most common reason for quarrels among sexual partners. That will usually result in violence against women, even during pregnancy (24, 37).

Among individual-level variables related to socio-ecological characteristics, the presence of marital conflict was identified as the most important determinant of IPV during pregnancy in our study. This finding is in line with other previous studies conducted in the country and abroad in which marital conflict was found to be an important predictor of IPV during pregnancy (5, 46, 47). This may be explained by the fact that women with marital conflicts are more likely to experience violence in a patriarchal society, like Ethiopia, where violence is perceived as a normal part of women's lives (5, 37).

Moreover, this study may enlighten the areas on which to focus in order to achieve the Sustainable Development Goals (SDGs) related to maternal health and violence against women. Regardless of goals, strategies, and programs designed at international (SDGs) and national levels, IPV during pregnancy is still unacceptably high in Ethiopia. Besides, both individual and contextual characteristics were identified as factors operating at different levels. This finding will help policymakers design strategies to combat those factors promoting women susceptibility to IPV, even during pregnancy. It can serve as evidence linking family planning services with the prevention of IPV during pregnancy. It may also highlight the importance of including IPV screening tools as an essential part of routine maternal health care services at the national level. Finally, the study can help the Ethiopian government design and implement programs that facilitate women's empowerment so that they can be prevented from experiencing violence before, during, and after pregnancy.

### Strength and limitations

This study has its own strengths, including using a large sample size that resulted in high power for the analysis. A strong statistical model (a multilevel model) was used to handle clustering effects. It also includes using a standardized and validated instrument from the WHO multi-country study on VAW. This study may have its limitations, which should be considered while interpreting the findings. The cross-sectional nature of the study might make it difficult to ascertain the temporal relationship. The sensitive nature of IPV might have introduced social desirability bias, recall bias, and non-disclosure, which might lead to under-reporting.

## Conclusions

This study revealed that the prevalence of intimate partner violence among pregnant women in the study area was high. The study also indicated that both community-level and individual-level factors were identified as factors associated with IPV during pregnancy. Access to health facilities, women feeling isolated from the community, strict gender role differences, maternal education, maternal occupation, living with a partner's family, women's decision-making power, pregnancy intended by a partner, dowry payment, and the presence of marital conflict were identified as factors associated with IPV during pregnancy. Special emphasis has to be given to educating and empowering women using various organized methods. Since it is a multi-dimensional problem, multi-sectoral approach is more appreciated. It is also recommended to design and implement different mechanisms to mitigate problems correlated to the norms (related to dowry payment, gender role differences and living with a partner's family) and socio-cultural factors that promote IPV in society. Community health planners and workers should design health promotion programs and create awareness of the prevention of IPV during pregnancy.

## Data availability statement

The original contributions presented in the study are included in the article/supplementary material, further inquiries can be directed to the corresponding author.

## Ethics statement

The studies involving human participants were reviewed and approved by Research and Ethical Review Committee (RER) of the School of Public Health, the Institutional Review Board (IRB) of the College of Health Sciences, Addis Ababa University. Written informed consent to participate in this study was provided by the participants' legal guardian/next of kin.

## Author contributions

MU conceived the study, developed the proposal, conducted data collection and analysis, and drafted the manuscript. AA and AY were involved in the proposal development, fieldwork supervision, data analysis, writing up, and critical reviewing of the manuscript. All authors contributed to the article and approved the submitted version.
